# Non-pharmaceutical interventions in response to the COVID-19 pandemic in 30 European countries: the ECDC–JRC Response Measures Database

**DOI:** 10.2807/1560-7917.ES.2022.27.41.2101190

**Published:** 2022-10-13

**Authors:** Lorenzo Lionello, Debora Stranges, Tommi Karki, Emma Wiltshire, Chiara Proietti, Alessandro Annunziato, Josep Jansa, Ettore Severi, on behalf of the ECDC–JRC Response Measures Database working group

**Affiliations:** 1 European Centre for Disease Prevention and Control, Stockholm, Sweden; 2 Joint Research Centre, European Commission, Ispra, Italy; 3 Additional members of the ECDC–JRC Response Measures Database working group are listed under Acknowledgements

**Keywords:** COVID-19, Non-pharmaceutical interventions, Public health policy, response measures, European Union/European Economic Area

## Abstract

In response to the COVID-19 pandemic, the European Union/European Economic Area (EU/EEA) countries implemented a wide set of non-pharmaceutical interventions (NPIs), sometimes with limited knowledge on their effect and impact on population. The European Centre for Disease Prevention and Control (ECDC) and the European Commission’s Joint Research Centre (JRC) developed a Response Measures Database (ECDC–JRC RMD) to archive NPIs in 30 EU/EEA countries from 1 January 2020 to 30 September 2022. We aimed to introduce a tool for the wider scientific community to assess COVID-19 NPIs effect and impact in the EU/EEA. We give an overview of the ECDC–JRC RMD rationale and structure, including a brief analysis of the main NPIs applied in 2020, before the roll-out of the COVID-19 vaccination campaigns. The ECDC–JRC RMD organises NPIs through a three-level hierarchical structure and uses four additional parameters (‘status’, ‘implementation’, ‘target group’ and ‘geographical representation’) to provide further information on the implementation of each measure. Features including the ready-for-analysis, downloadable format and its agile taxonomy and structure highlight the potential of the ECDC–JRC RMD to facilitate further NPI analysis and optimise decision making on public health response policies.

## Background

The impact of the 2019 coronavirus disease (COVID-19) made 2020 one of the most defining years of the current century. As the severe acute respiratory syndrome coronavirus 2 (SARS-CoV-2) quickly spread across the world [1], saturating intensive care units’ capacity and placing healthcare systems under great strain [2], many governments responded with the implementation of different non-pharmaceutical interventions (NPIs) [3-9]. Since there was limited scientific knowledge regarding the effect of most of those measures, the NPIs that were introduced varied greatly across and within countries [3,8,10,11]. The lack of knowledge on evidence-based interventions has contributed to the creation of databases which allow for the collection of the NPIs implemented in the different areas and settings [12-14]. This perspective aims at introducing a tool for the scientific community and public health policy makers to assess effect and impact of COVID-19 NPIs in the EU/EEA by presenting the European Centre for Disease Prevention and Control (ECDC) and the European Commission’s Joint Research Centre (JRC) Response Measures Database (ECDC–JRC RMD), describing its structure and providing a rapid overview of the NPIs implemented by the 30 European Union and European Economic Area (EU/EEA) countries from 1 January to 31 December 2020. The ECDC–JRC kept recording NPIs implemented or modified in the EU/EEA from 1 January 2020 to 30 September 2022.

## The ECDC–JRC Response Measures Database

In March 2020, the ECDC–JRC RMD was initiated for the urgent need to (i) collect information to rapidly inform ECDC, JRC and the European Commission of the different NPIs implemented in the EU/EEA countries, (ii) describe the COVID-19 pandemic and the measures taken in response to it in the EU/EEA, (iii) support the modelling efforts in ECDC to issue projections on the pandemic trajectory in the EU/EEA and (iv) assess effectiveness and impact of the measures in place. In summary, the ECDC–JRC was created to understand how countries responded to the global emergency and learn lessons on their effect and impact for the current and future public health emergencies.

**Figure 1:**
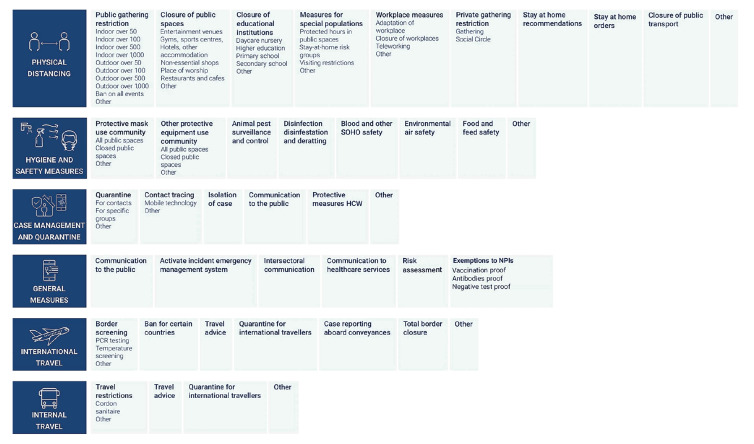
Complete hierarchical structure of the European Centre for Disease Prevention and Control–European Commission’s Joint Research Centre Response Measures Database, 30 European Union/European Economic Area countries, 1 January 2020–30 September 2022

HCW: healthcare worker; NPI: non-pharmaceutical interventions.

The ECDC–JRC RMD is organised in a hierarchical structure (Figure 1). The measures entered are recorded following a three-level coding system (Levels 1, 2 and 3). Level 1 represents six macro categories of interventions [15,16]: (i) case management and quarantine measures, (ii) general measures (emergency declarations, communications and risk assessments, NPI exemptions). (iii) hygiene and safety measures, (iv) internal (domestic) travel measures, (v) international (cross-border) travel measures and (vi) physical distancing measures. NPIs are further categorised by two additional levels (Level 2 with 41 categories and Level 3 with 51) to increase specificity and provide a more detailed explanation on the aim of the measure. A total of 79 individual combinations are possible (Figure 1).

Non-pharmaceutical interventions are classified with a three-coded system: Level 1 measures are shown in the left-most column (dark blue), Level 2 measures are represented by headings in bold, and Level 3 measures are listed below each Level 2 measure.

Four additional parameters are available in the ECDC–JRC RMD to provide further information on the implementation of each measure. These are ‘status’, ‘implementation’, ‘target group’ and ‘geographical representation’.

The ‘status’ parameter (i.e. mandatory, voluntary or not available) represents the rigour of the legal basis behind the measure. The ‘implementation’ parameter (i.e. full or partial) indicates when a measure allows for exemptions or not: full measures affect the whole specified target population with no exception, while partial measures might have some exemptions that exclude a part of the target population or have exceptions to the restriction. The ‘target’ parameter represents a list of indicative population groups that the NPI is directed towards. Finally, all measures have a ‘geographic representation’ with detailed national, regional, and local implementations coded. The subnational division for each country has been aligned as much as possible with the geographical and administrative granularity of the epidemiological data collected by ECDC to allow for NPI effect analysis.

A new record is added to the database each time one of the parameters described above is changed. All information is gathered from official public sources. For the taxonomy and a more detailed explanation of each coding pathway, please visit the terminology page of the public database (https://www.ecdc.europa.eu/en/publications-data/response-measures-database-rmd).

## Data collection

Starting in March 2020, a dedicated team at ECDC collected data, fortnightly, on NPIs implemented in the 30 EU/EEA countries. At the beginning of the data collection, a retrospective search was carried out to include measures implemented from January to March 2020. To ensure consistency in the data collection, each data collector had to refer to the same coding pathways each time they entered new measures. After each round of updates, to ensure good data quality, an internal team of reviewers at ECDC cross-checked all new entries to guarantee that they had been correctly recorded. A case-by-case approach was used to review and resolve every ambiguous coding choice. In addition to the fortnight data validation, ad hoc data quality checks have been regularly performed to improve both the database data quality and the consistency of the information between and within countries. Such data quality checks (e.g. reviewing all information within one country and all information on the same measure in different countries) are performed using a standardised protocol internal to ECDC.

## Overview of the non-pharmaceutical interventions implemented in 2020

We present a brief descriptive analysis of the measures captured in the ECDC–JRC RMD from 1 January to 31 December 2020, to (i) offer a snapshot of the records that the database supplies, (ii) help possible RMD users understand the scope of the database and (iii) give a sense of the measures in place before the COVID-19 vaccine was made available in EU/EEA countries. The measures are presented by coding category, period and length of implementation. For selected NPI groups, the median length of implementation and related interquartile range (IQR) are also presented. Records that were still active on 31 December 2020 are included, but for the purpose of this paper, they are considered only to last until 31 December 2020. All data analyses and visualisations were done using R version 4.0.3 [17].

From 1 January to 31 December 2020, 3,198 individual NPIs implemented in 30 EU/EEA countries were recorded in the ECDC–JRC RMD. Of these (n = 314; 10%) were still active on 31 December 2020, meaning they either had no recorded end date, or had an end date in 2021 or 2022. The majority of reported NPIs were related to physical distancing measures (n = 2,198; 69%), followed by measures restricting international travel (n = 280; 9%), which had a high degree a heterogeneity among different EU/EEA countries [18], and hygiene and safety measures (n = 222; 7%).

**Figure 2:**
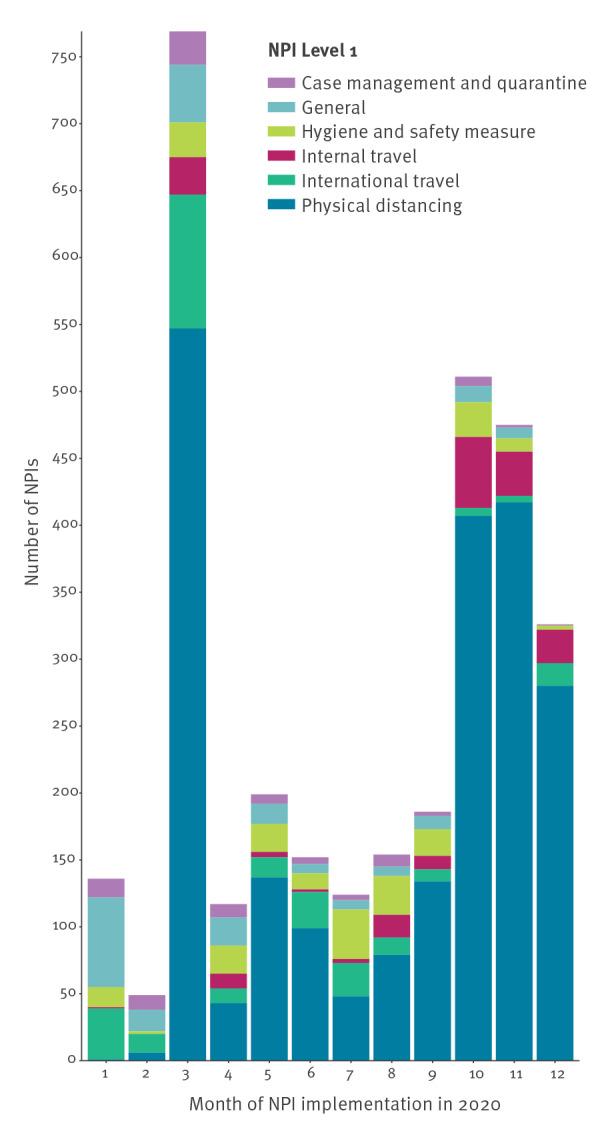
Distribution of Level 1 non-pharmaceutical interventions by month of implementation, European Centre for Disease Prevention and Control–European Commission’s Joint Research Centre Response Measures Database, 30 European Union/European Economic Area countries, 1 January–31 December 2020 (n = 3,198)

In 2020, the month with the highest number of recorded NPIs was March 2020 (n = 769; 24%) followed by October 2020 (n = 511; 16%) (Figure 2).

NPI: non-pharmaceutical interventions.

The y axis represents the sum of NPIs implemented by the 30 EU/EEA countries during the month specified.

The median length of implemented measures was 5 weeks (35 days; IQR: 12–74) with great heterogeneity in the length of NPIs both within and between Level 1 categories.

**Figure 3:**
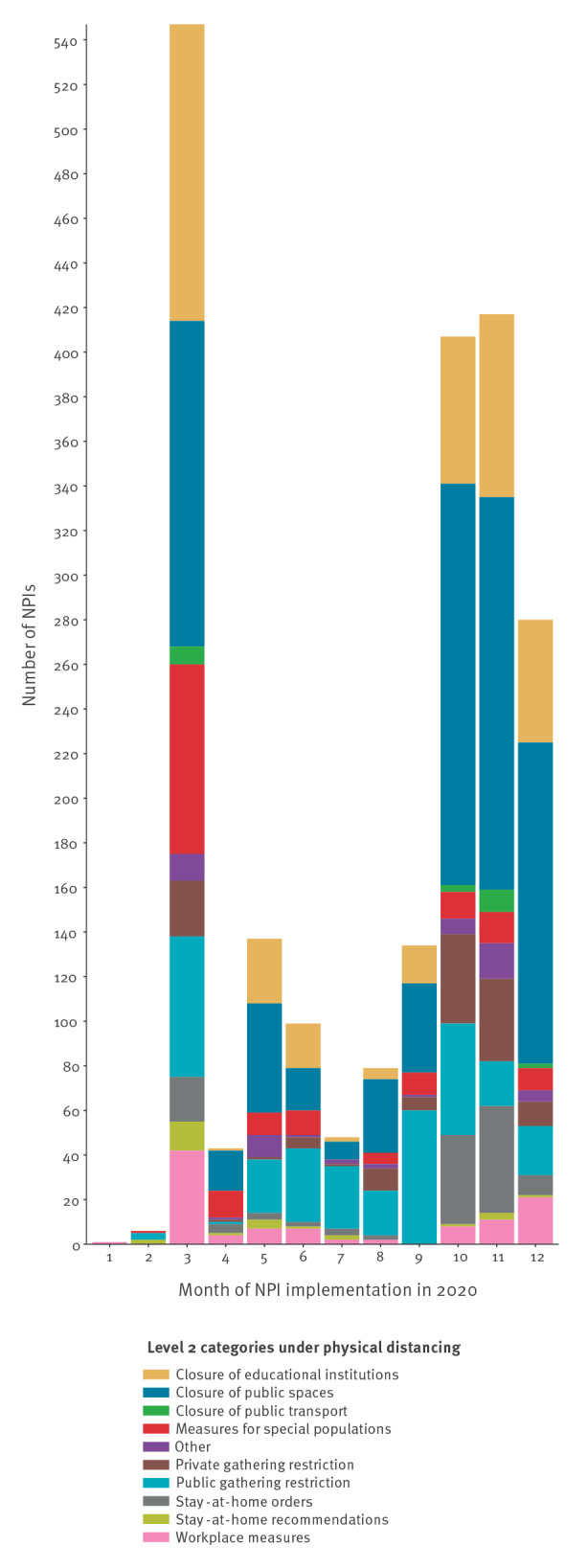
Distribution of Level 2 physical distancing non-pharmaceutical interventions by month of implementation, European Centre for Disease Prevention and Control–European Commission’s Joint Research Centre Response Measures Database, 30 European Union/European Economic Area countries, 1 January–31 December 2020 (n = 2,198)

Figure 3 shows the Level 2 disaggregation of physical distancing measures, which is the most prominent category in the database. National stay-at-home orders (coded as ‘full’ when the order was about staying at home all day) and curfews (coded as ‘partial’ when the order was about staying at home only at night) were introduced in 20 EU/EEA countries in 2020. The longest ‘full’ stay-at-home order in the database was nearly 8 weeks (55 days) long; the stay-at-home orders median duration was about 3 weeks (22 days; IQR: 14–40). Curfews (coded as partial stay-at-home orders in the ECDC–JRC RMD) became more prominent in the last quarter of 2020, and these had a median duration of nearly 4 weeks (26 days; IQR: 16–57), with the longest curfew in 2020 lasting 10.5 weeks (73 days).

NPI: non-pharmaceutical interventions.

The y-axis represents the sum of physical distancing NPIs implemented by the 30 EU/EEA countries during the month specified. Stay-at-home orders include both orders to stay home all the time (coded as ‘full’ stay-at-home orders) and orders to stay home only at night (i.e. curfews; coded as ‘partial’ stay-at-home orders).

School closures are also included in the Level 2 physical distancing category. All 30 EU/EEA countries introduced some form of educational institution closure in 2020. The longest school closure of any type of educational institution was a full higher education institution closure, which lasted about 41 weeks (288 days, including regular summer holidays). The median length of closure in educational institutions was nearly 5 weeks (33 days; IQR: 13–67). Secondary schools were the most affected, with a total of 148 records regulating in-presence learning in some way (36% of the NPIs in this category, n = 410). This was followed by higher education (n = 116; 28%) and primary schools (n = 83; 20%). Of the orders restricting physical presence in educational settings, 32% of all school interventions (n = 133) were introduced in March 2020, while 50% of all school interventions (n = 203) were implemented between October and December 2020.

Face mask use in public spaces is one of the most common measures in the database. The first mask requirement was introduced on 28 February 2020 and 27 countries followed by introducing some form of mandatory mask use in indoor spaces (including public transport) and 28 countries introduced mandatory use of face masks in all public spaces. Face mask orders (in all indoor and outdoor public spaces) had a median length of about 6 weeks (44 days; IQR: 17–83).

## Current and future implications

The ECDC–JRC RMD offers a rich archive of NPIs taken by EU/EEA countries during 2.5 years of public health emergency and aims to provide a thorough and complete source of NPIs introduced. The ECDC–JRC RMD can be exported in a ready-for-analysis format. It also makes use of a taxonomy and structure that can be of help for those countries that are either analysing NPI effectiveness and impact, or retrospectively analysing the NPIs implemented during the COVID-19 pandemic to build national databases.

Despite the systematic and extensive data validation and data quality checks performed after each update, the ECDC–JRC RMD presents some limitations. Firstly, measures across the database may be subject to personal interpretation and bias in coding practices, resulting in variations between countries with similar measures. While the NPIs recorded in the database include a wide variety of possible measures that have been introduced, this database is by no means a complete representation of all the actions taken by the countries monitored. For a more thorough explanation on the type of measures that the database intended to record, please refer to a public version of the database [19].

Secondly, the primary level of the NPIs collected in the RMD is the national level. Subnational data are also collected; however, there is variation in the completeness of the subnational data collected. While some countries have a wealth of subnational measures recorded, for others it was not possible to collect a high degree of subnational information because of large heterogeneity of reporting across the country and how often the measures change. This discrepancy is, in part, also due to the administrative structures that EU/EEA countries adopted in the public health emergency: highly independent administrations were much harder to track, so a generalised national approach was adopted in these cases (e.g. Germany).

Thirdly, across Europe, interventions have changed in intensity and scope according to the period in which they were introduced, and the ECDC–JRC RMD has tried to control for these characteristics across time by including the parameter of full or partial implementation. However, this remains an approximation of the actual mechanisms at play. Finally, the database is constantly evolving and is periodically retrospectively revised to improve its quality and accuracy. While these revisions do not have a substantial impact on the overall database, some of the descriptive statistics presented above might not be reproducible because of changes happening after the time of publication.

Prior to the current pandemic, our understanding of NPIs at global scale was primarily based on outdated observations and insufficient data. In part for this reason, including NPIs in preparedness and response plans was complex [10,20]. Despite its limitations, the ECDC–JRC RMD provides a rich collection of data and the opportunity for future research and analysis. Layered over epidemiological data, the information provided by the database can help assess the effectiveness of the interventions to guide decision-making. In addition, the ECDC–JRC RMD is not the only database codifying NPIs across Europe, and combining the data gathered here with other similar works can give researchers a unique opportunity of analysis, with each database complementing the shortcoming of the others [12-14].

## Conclusions

Since spring 2020, ECDC has used the ECDC–JRC RMD information to support modelling projections and assess the effect for certain NPIs, such as stay-at-home orders, public gathering cancellations and school closures. Information from the ECDC–JRC RMD is currently used for in-action and after-action reviews and for the preparation of lessons learnt on the current emergency. The ECDC–JRC RMD is a tool made available to the scientific community to assess NPIs effectiveness and impact during this unprecedented pandemic. Such a tool can be used to better tailor responses to public health emergencies, so as to maximise the effect of NPIs during public health emergencies while decreasing the associated negative impact on the population. The study of the data on NPIs will contribute to efforts to re-shape the future of public health preparedness and response in the years to come.
